# 3D occlusal changes of upper first molars after rapid maxillary expansion on permanent versus deciduous teeth: a retrospective multicenter CBCT study

**DOI:** 10.1186/s40510-023-00476-1

**Published:** 2023-07-31

**Authors:** Marco Serafin, Rosamaria Fastuca, Piero Antonio Zecca, Manuel Lagravère, Alberto Caprioglio

**Affiliations:** 1 Department of Biomedical Sciences for Health, University of Milan, Milan, Italy; 2 Varese, Italy; 3 Department of Medicine and Surgery, School of Medicine, University of Insubria, Varese, Italy; 4 Orthodontics Department, Faculty of Medicine and Dentistry, University of Alberta, Edmonton, Canada; 5 Department of Biomedical, Surgical and Dental Sciences, Section of Orthodontics, University of Milan, Milan, Italy; 6 Fondazione IRCCS Cà Granda, Ospedale Maggiore Policlinico, Milan, Italy

**Keywords:** CBCT, Deciduous teeth, Maxillary first molar, Occlusion, Rapid maxillary expansion

## Abstract

**Background:**

The purpose of this study was to compare the three-dimensional dental changes for the maxillary first molars and the overall skeletal effects achieved after expansion between the rapid maxillary expansion (RME) appliance attached to two different anchor units, the maxillary deciduous molars and the maxillary permanent first molars.

**Methods:**

Patients were retrospectively selected according to the anchorage unit used for RME: deciduous upper second molars (RME-E group; 10 M, 10 F; mean age 8.4 ± 1.1 years) and first upper permanent molars (RME-6 group; 10 M, 10 F; mean age 12.6 ± 1.8 years). CBCT scans were obtained before treatment start (T1) and after retention and removal of the expander (T2). Images were analyzed using a new three-dimensional intra-hemi-maxillary reference system. 3D landmarks were marked to calculate all changes on maxillary first permanent molars; mesio-distal and buccal-lingual inclination and rotation, as well as intermolar and interforaminal distances were calculated. The Wilcoxon test was used to compare within-group changes, whereas the Mann–Whitney test was used to compare between-group differences, with the significance level set at 0.05.

**Results:**

In the RME-E group, significant distorotation and lingual inclination of the first permanent molars at T2 were observed (*p* < 0.01); in the RME-6 group, only the buccolingual inclination of the crossbite side after RME was resulted statistically significant (*p* < 0.01). In both groups, intermolar and interforaminal values increased significantly (*p* < 0.01). Intergroup analysis showed a significantly higher distorotation and reduced buccal inclination of maxillary first permanent molars in the RME-E group after RME (*p* < 0.01).

**Conclusions:**

RME is effective in treating maxillary transverse hypoplasia; RME anchored too deciduous teeth spontaneously reduces buccal inclination and increases distorotation of maxillary first permanent molars, whereas anchorage to permanent molars is associated with increased buccal inclination, albeit with little clinical significance.

## Background

Transverse maxillary deficiency is one of the most common clinical conditions nowadays, which may be associated with crossbite in the posterior region and crowding in the anterior one. When skeletal transverse hypoplasia of maxillary arch is diagnosed, orthopedic skeletal expansion is suggested [1]. This treatment is especially recommended in growing patients before the craniofacial sutures are completely calcified, so that the appliance can act on the maxillary sutured and skeletal asymmetries do not develop [2]. Although several expansion protocols have been proposed, rapid maxillary expansion (RME) is still one of the most common early treatments performed by clinicians to release the posterior crossbite and gain additional space in the maxillary arch [3]. In addition, it has been suggested that this treatment can effectively prevent and improve functional problems associated with abnormalities of the breathing pattern [2].

RME was first described by Angell in 1860 [4] and the fixed maxillary expander is still the most commonly used appliance, usually anchored to permanent or deciduous teeth, with different protocols depending on the frequency of activations, the amount of force applied, the duration of treatment, and the patient’s age [5]. Traditionally, RME treatment should result in minimal tooth movement and maximum skeletal movement, since the forced generated by the appliance should be transmitted directly to the sutures thanks to the hyalinization of the periodontal ligament of the anchor teeth [6]. The RME increases the transverse dimensions of the maxilla mainly by separating of the two halves, followed by buccal movement of the posterior teeth and alveolar processes [7]. Due to the high forces that can occur during RME, special attention has been paid to the anchorage system, as the maxillary expander was previously anchored to the maxillary fist permanent molars, resulting in collateral damage such as exostosis, pulp stones, root resorption, and periodontal damage [8]. In fact, the expansion forces, that cause buccal tipping, can lead to a reduction in the level of the alveolar bone crest, bone dehiscence, and gingival recession [9].

To avoid these undesirable effects on the permanent teeth and to achieve a more physiologic tissue response, RME in the mixed dentition can be achieved by using deciduous teeth as anchorage units for expansion, which could be helpful to avoid resorption, bone loss, gingival recession, and white spot lesions in the permanent dentition [10]. During maxillary expansion on deciduous teeth, a transverse increase in skeleton was observed, and permanent molars are indirectly expanded [11, 12]; moreover, changes in the mandible can also be expected [1].

The effectiveness of RME on skeletal and dental structures was previously determined from dental casts, lateral and posteroanterior cephalograms, and occlusal radiographs. Previous studies of the effects of RME treatment were performed using 2D radiographic analyzes, which have their limitations and do not allow accurate assessment of the affected structure without structural overlap [13]; furthermore, 2D imaging does not represent the totality of 3D structures [14]. Since the introduction of the Cone beam Computed Tomography (CBCT) in dentistry atin the late 1990s, it has been increasingly used for orthodontic diagnosis, treatment planning and research [15]. With the availability of CBCT, many cephalometric limitations have been eliminated. The use of novel imaging techniques for 3D imaging allows high precision and accuracy with minimal image distortion when measuring linear and angular parameters at skeletal or dental landmarks, regardless of the movement of plane references caused by orthopedic distraction, e.g., maxillary expansion creating a suture opening in a vertical and sagittal pyramidal pattern [16].

Since the maxillary permanent molars are not constrained in the bands of the appliance when deciduous teeth are anchored in the RME, they are free to adapt to the best occlusal situation of the patient and spontaneous movement of these teeth have been observed as rotation and changes in bucco-lingual inclination [17, 18]. CBCT cloud be a great tool to explore the three-dimensional changes that may occur in the crowns and roots of maxillary first molars when the expander is anchored to the deciduous teeth and studies that evaluate all the movements in the same group of patients have not been published.

The main hypothesis is that dental anchorage on deciduous teeth can facilitate a natural three-dimensional decompensation of the first permanent molar, which can result in a distorotation to a favorable Class I molar relationship and a reducing of torque without compromising periodontal health. Therefore, the aim of the present study was to evaluate the three-dimensional skeletal changes and dental changes of the maxillary first permanent molars in patients who underwent RME with anchorage to deciduous versus permanent teeth.

## Materials and methods

### Sample selection

The present study was designed retrospectively. Ethical approval was obtained from the Committee of the University of Milan (Milan, Italy; approval number: 573/15) and the University of Alberta (Edmonton, Canada; approval number: Pro00013379), and procedures were in accordance with the Declaration of Helsinki of the World Medical Organization. Signed informed consent for scientific purposes was obtained before the start of treatment.

In this retrospective study, we enrolled patients who required orthodontic planning and had no history of orthodontic treatment. Our sample consisted of patients selected from the Department of Orthodontics at the University of and the Department of Dentistry at the University of. All subjects had RME on deciduous molars (RME-E) or permanent molars (RME-6) and were selected based on the following inclusion criteria: good general health, mixed dentition with fully erupted permanent first molars, maximum age < 15 years, skeletal transverse discrepancy with unilateral posterior crossbite before treatment, good periodontal health, no caries with 2 or more involved surfaces, and available initial and final diagnostic records including good quality CBCT scans without motion artifacts. The exclusion criterion was poor quality CBCT scans or missing records. All patients with RME in the mixed dentition (RME-E), in whom the appliance was anchored to the deciduous teeth were treated at the Department of Orthodontics, University of. All patients with RME in the permanent dentition (RME-6), in whom the appliance was anchored to permanent maxillary first molars, were treated in the Department of Orthodontics, University of. Each patient in both groups received 2 CBCT scans, before starting treatment with RME (T1) and within 30 days after removal of the expansion appliance (T2). Table 1 describes the samples included in the present study.

**Table 1:** Descriptive analysis of the RME-E and RME-6 groups

	RME-E Group	RME-6 Group
Total sample	20	20
Male/female	10/10	10/10
Mean age (Years)	8.4 ± 1.1	12.6 ± 1.8
Range (Years)	6.9–10.9	6.7–14.9

The RME appliance used for RME-E was a tooth-supported hyrax expansion appliance with bands on the deciduous second molars, extension arms bonded to the deciduous canines, and no distal extension arm on the permanent first molars. The expansion appliances were cemented with glass ionomer cement according to the manufacturer’s instructions. The expansion screw was initially activated twice (0.45-mm initial activation). Later, the patients’ parents were instructed to activate the screw twice per day (0.45-mm activation per day) starting the day after placement. Maxillary expansion was performed until dental overcorrection, defined as the point where the palatal cusp of the maxillary permanent first molars occludes on the inner slope of the buccal cusp of the mandibular permanent first molars. The screw was activated by 30 ± 3 turns in the RME-E group (mean opening, 6.3 mm). After the active expansion treatment, the appliance was passively held in position for 6 months. During this time, no patient had any other orthodontic treatment. CBCT scans (i-CAT, 120 kV, 3.8 mA, 30 s; Imaging Sciences International, Hatfield, Pa) were performed before and after treatment, immediately after appliance removal (mean interval, 9 ± 1 months). The appliance used for group RME-6 was a tooth-supported Hyrax expansion appliance with bands on the permanent first molars and first premolars. The expansion screw was activated twice daily (0.45 mm per day activation) until overcorrection of the teeth was achieved. After active expansion treatment, the appliance was passively kept in place for 6 months. CBCT scans (3G Newtom, 110 kV, 6.19 mA, 9 s; Aperio Services, Verona, Italy) were performed before and after treatment, immediately after the removal of the appliance (mean interval, 8.6 ± 2 months). The screw was activated in the RME-6 group with 20 ± 5 turns (mean opening, 4.7 mm).

### Image analysis

T1 and T2 CBCT data were saved in DICOM format and converted to a volumetric image. The DICOM files were then processed with dedicated software (Mimics version 19.0; Materialise NV, Leuven, Belgium) to orient and standardize them. Sagittal, axial, and coronal slices, as well as 3D image reconstruction, were used to determine the position of the landmarks; the 3D landmarks are defined in Table 2.

**Table 2:** Landmarks, mathematical reconstruction and measurements of the analyzed data

*3D landmark position*	
Anterior nasal spine (ANS)	Most anterior point of the ANS
Posterior nasal spine (PNS)	Most posterior point of the PNS
Greater palatine foramen (GPF)	Geometric center of the GPF
Mesial pulp horn (MPH)	Highest occlusal point of the MPH of U6
Distal pulp horn (DPH)	Highest occlusal point of the DPH of U6
Palatal pulp horn (PPH)	Highest occlusal point of the PPH of U6
Mesial root apex (MRA)	Geometric center of the MRA of U6
Distal root apex (DRA)	Geometric center of the DRA of U6
Palatal root apex (PRA)	Geometric center of the PRA of U6
*3D reconstruction and rendering*	
First permanent Molar (U6)	Rendered pyramid trunk solid formed by MPH-DPH-PPH base and MRA-DRA-PRA base
First molar center (U6-C)	Center of mass of U6
First molar axis (U6-A)	Line passing through the centroid of the upper and lower bases of U6
Pulp horn line (PH-L)	Line passing through MPH-PPH
Hemimaxillary plane (Hmx-P)	Plane passing through the ANS-PNS-GPF
Sagittal plane (x-P)	Plane passing through PNS and ANS, perpendicular to the respective right or left Hmx-P
Coronal plane (y-P)	Plane passing through PNS, perpendicular to right or left Hmx-P and x-P, respectively
Axial plane (z-P)	Plane passing through the PNS and parallel to the right or left Hmx-P
*Hemimaxillary and maxillary measurements*	
Mesiodistal angulation (MD-angulation)	Angle between U6-A and coronal plane
Buccolingual inclination (BL-inclination)	Angle between U6-A and sagittal plane
Rotation (Rot)	Angle between PH-L and sagittal plane
Intermolar distance (Intermolar)	Sum of distances between right and left U6-C to x-P
Interforaminal distance (Interforaminal)	Sum of distances between right and left GPF to x-P

**Fig. 1:**
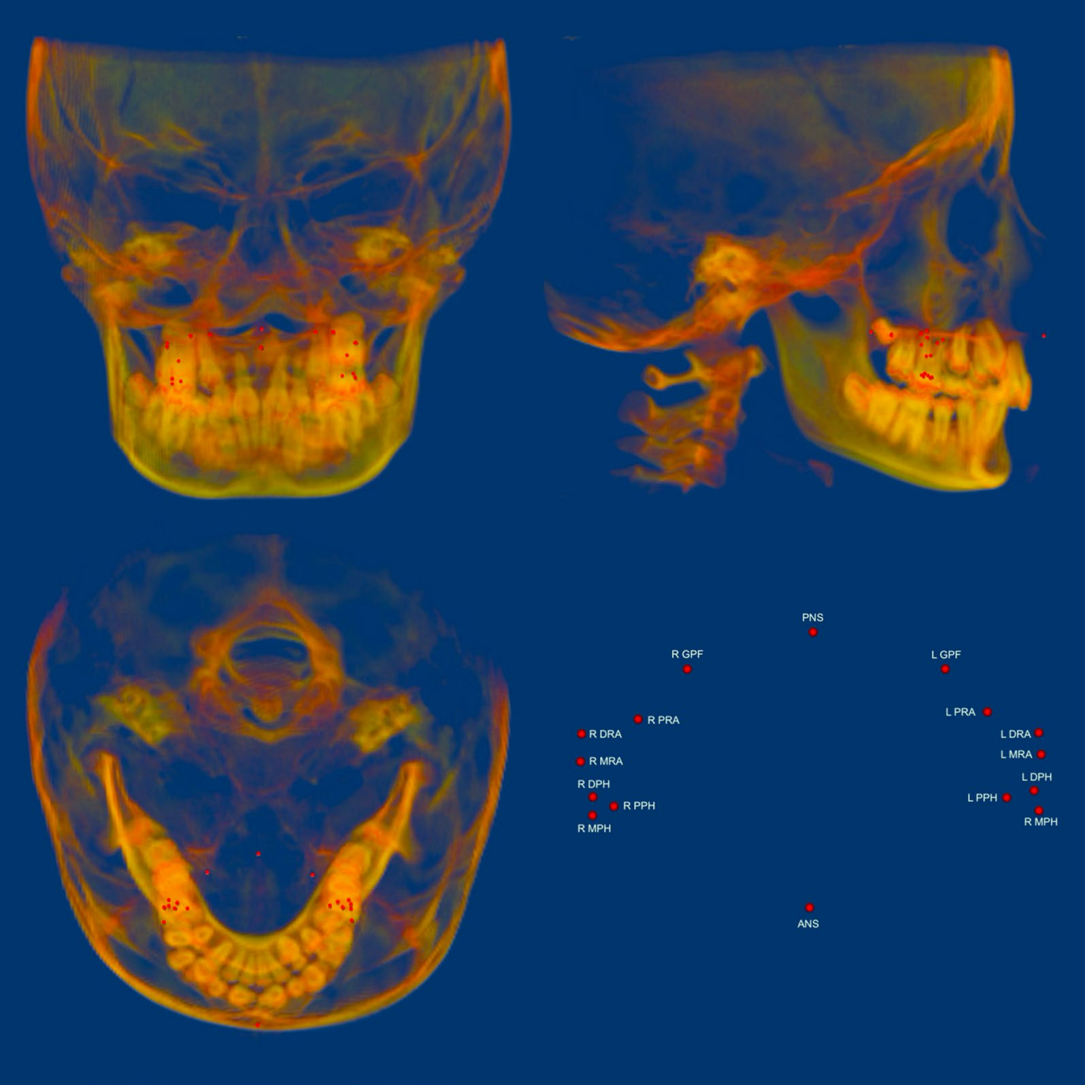
Graphical representation of the described landmarks

**Fig. 2:**
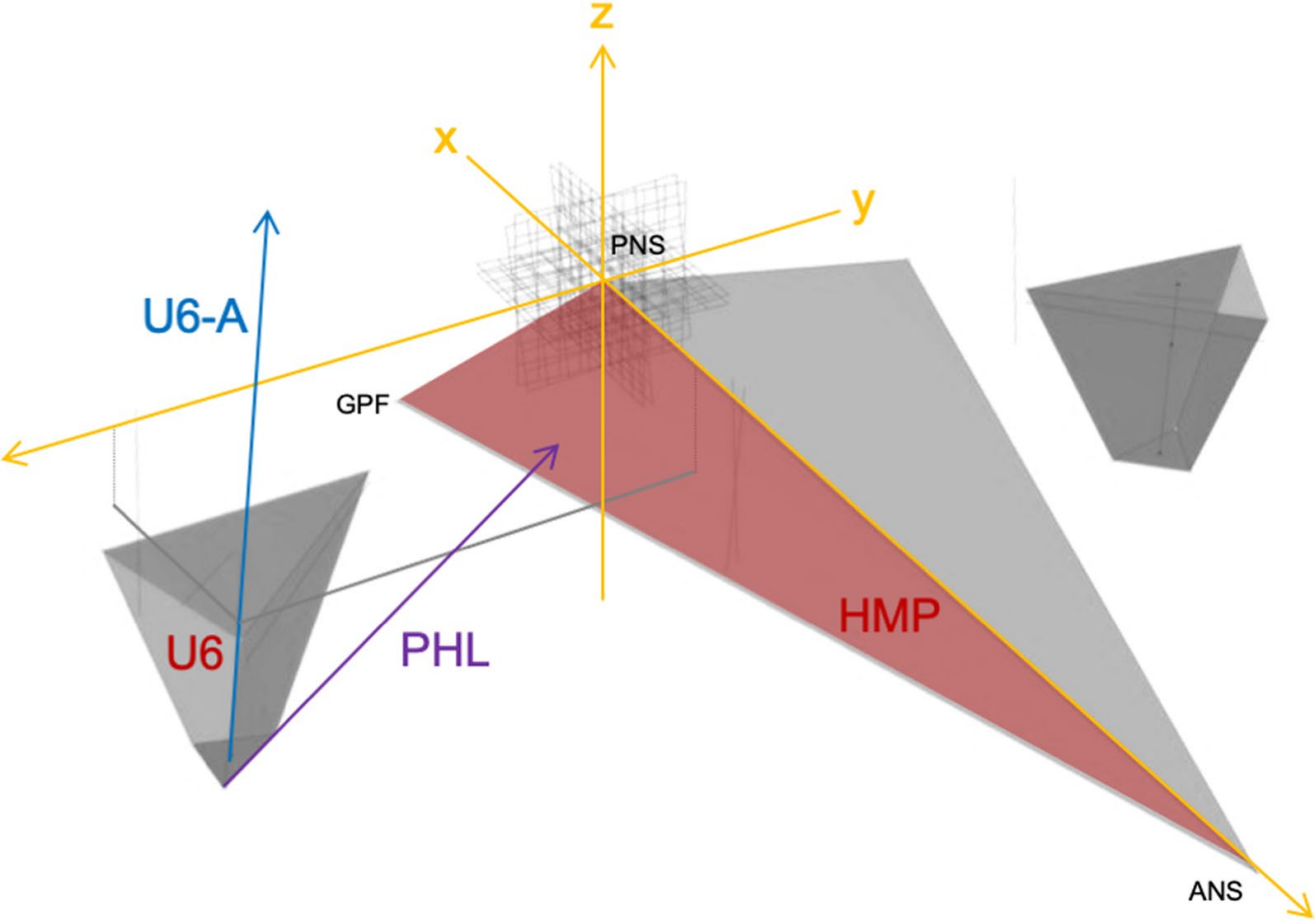
Graphic representation of the mathematical model used for the analysis of linear and angular measurements

A three-dimensional reference system was used in the present study. The landmarks found on the CBCT were exported and then processed in CAD software (Grasshopper plugin; Rhinoceros 3D, Version 6.0. Robert McNeel & Associates, Seattle, WA) to construct the reference planes; Fig. 1 represents a graphic reconstruction of the positioning of landmarks. As described in Table 2, a 3D volume and surface rendering of the upper first molar and corresponding hemimaxilla, respectively, was created. A new reference point was used: PNS was set as the x, y, and z origin and the corresponding coordinate planes emanating from it in the hemimaxillary plane (x: sagittal; y; coronal; z: axial); the resulting axes and planes and their orientation are described in Table 2. In addition, all monolateral data were split between the side with crossbite and the side without it. As described in Table 2, the monolateral linear measurements are thought of as Euclidean distance, i.e., the orthogonal distance of a reference point to a plane, while the angular measurements are formed by the intersection of an axis and a reference plane; consequently, the bilateral linear measurements are obtained by summing the right and left monolateral measurements. Finally, the software analysis was performed to obtain the linear and angular measurements listed in Table 2; the graphic representation of the mathematical model used for the analysis of linear and angular measurements is shown in Fig. 2.

### Statistical analysis

SPSS software (version 22.0; IBM Corp., Armonk, NY, USA) was used for statistical analysis. The Shapiro-Wilk test showed a nonnormal distribution of the data; therefore, nonparametric tests were used for statistical analysis. Means and standard deviations were calculated for both groups. The Mann–Whitney U test was used to compare the baseline forms of the 2 groups. Each group was analyzed as a whole without stratification by sex. Measurements in the RME-E group were compared with those in the RME- 6 group. The Wilcoxon test was used to compare changes between the time points in the same group, and the Mann–Whitney U test was used to compare differences between groups.

### Method error

To calculate method error five randomly selected CBCTs from each group were retraced at 2-months interval by a trained operator (MS). Using the Mann–Whitney U test, no significant differences were found in landmark placement, tracing, and measurement of the 11 variables, with the significance level set at *p* < 0.05. The reported average error was 0.16 mm, with a minimum error of 0.04 mm and a maximum error of 0.37 mm.

## Results

The statistical analysis performed on the baseline forms showed that the values of buccolingual inclination were statistically significant between the RME-E and RME-6 groups, both between the crossbite side (*p* = 0.001) and the non-crossbite side (*p* = 0.002); all others variables tested showed no statistical significance, as shown in Table 3. The results of the within-group comparisons for the RME-E and RME-6 groups are shown in Table 4.

**Table 3:** Mean and standard deviation (SD) of intergroup RME-E and RME-6 analysis at T1

Measurements		RME-E T1		RME-6 T1		*p*-value
		Mean	SD	Mean	*SD*	
MD-angulation (°)	Crossbite	7.67	4.11	6.22	3.39	0.334
	Non-crossbite	7.47	4.03	5.42	4.09	0.067
BL-inclination (°)	Crossbite	13.23	5.32	6.16	4.98	0.001*
	Non-crossbite	17.36	5.42	10.59	5.68	0.002*
Rot (°)	Crossbite	53.77	7.61	54.03	8.70	0.841
	Non-crossbite	54.15	8.83	54.70	7.86	0.665
Intermolar (mm)	–	43.51	2.94	41.93	3.06	0.301
Interforaminal (mm)	–	26.38	2.77	27.50	1.52	0.089

^*^p < 0.05

**Table 4:** Mean and standard deviation (SD) of RME-E and RME-6 intra-group and inter-group comparison between T1 and T2; (–) represents distal angulation and lingual inclination; (+) represents distal rotation

Measurements		RME-E ΔT2-T1	RME-6 ΔT2-T1					ΔE-6	*p*
		Mean	SD	*p*	Mean	SD	*p*		
MD-angulation (°)	Crossbite	0.01	2.78	0.526	− 0.61	1.86	0.111	0.62	0.594
	Non-crossbite	0.76	3.03	0.145	− 0.05	2.10	0.798	0.81	0.182
BL-inclination (°)	Crossbite	− 2.67	5.93	0.073	2.39	2.86	0.017*	− 5.06	0.003*
	Non-crossbite	− 3.49	5.26	0.001*	0.57	3.94	0.334	− 4.06	0.006*
Rot (°)	Crossbite	8.61	6.65	0.000*	2.45	6.18	0.191	6.16	0.006*
	Non-crossbite	8.12	8.26	0.001*	0.32	4.98	0.755	7.80	0.002*
Intermolar (mm)	–	3.24	1.20	0.000*	2.81	1.80	0.001*	0.43	0.317
Interforaminal (mm)	–	2.07	1.22	0.000*	1.79	1.07	0.001*	0.28	0.594

^*^p < 0.05

In the RME-E group, treatment resulted in significant distorotation of the upper first molars in the crossbite (8.61 ± 6.65°; *p* < 0.001) and non-crossbite side (8.12 ± 8.26°; *p* = 0.001). The buccolingual inclination decreased significantly (3.49 ± 5.26°; *p* = 0.001) in non-crossbite side. Statistically significant was also the increase in intermolar distance (3.24 ± 1.20 mm; *p* < 0.001) and interforaminal distance, which increased by 2.07 ± 1.22 mm (*p* < 0.001). The other parameters tested showed no significant difference between T1 and T for the RME-E group. The RME-6 group showed a significant increase in intermolar and interforaminal distances, which changed by 2.8 ± 1.8 mm and 1.8 ± 1.1 mm, respectively (*p* = 0.001). Other variables remained stable in the interval between T1 and T2 with the exception of buccolingual inclination for the crossbite side, which significantly increased by 2.39 ± 2.86° (*p* = 0.017). In Table 4, the multiple comparison between the means of the two groups after treatment was also resumed. Statistical analysis showed significant differences between crossbite and non-crossbite sides for rotational and buccolingual inclination variables between the groups, but no significant changes in mesiodistal angulation (*p* > 0.05). Distorotation of the upper first permanent molars was significantly higher in the RME-E group than in the RME-6 group. Comparison between groups showed similar and statistically significant differences between sides with (*p* = 0.006) and without crossbite (*p* = 0.002). In addition, the RME-E group showed significant lingual inclination of the upper molars compared to the RME-6 group, which, on the contrary, showed buccal inclination; statistically significant were the differences in buccolingual inclination in both crossbite (*p* = 0.003) and non-crossbite (*p* = 0.006) groups. Transversal analysis revealed in a very small clinically and statistically significant in Intermolar (*p* = 0.317) and Interforaminal (*p* = 0.594) measurements.

**Fig. 3:**
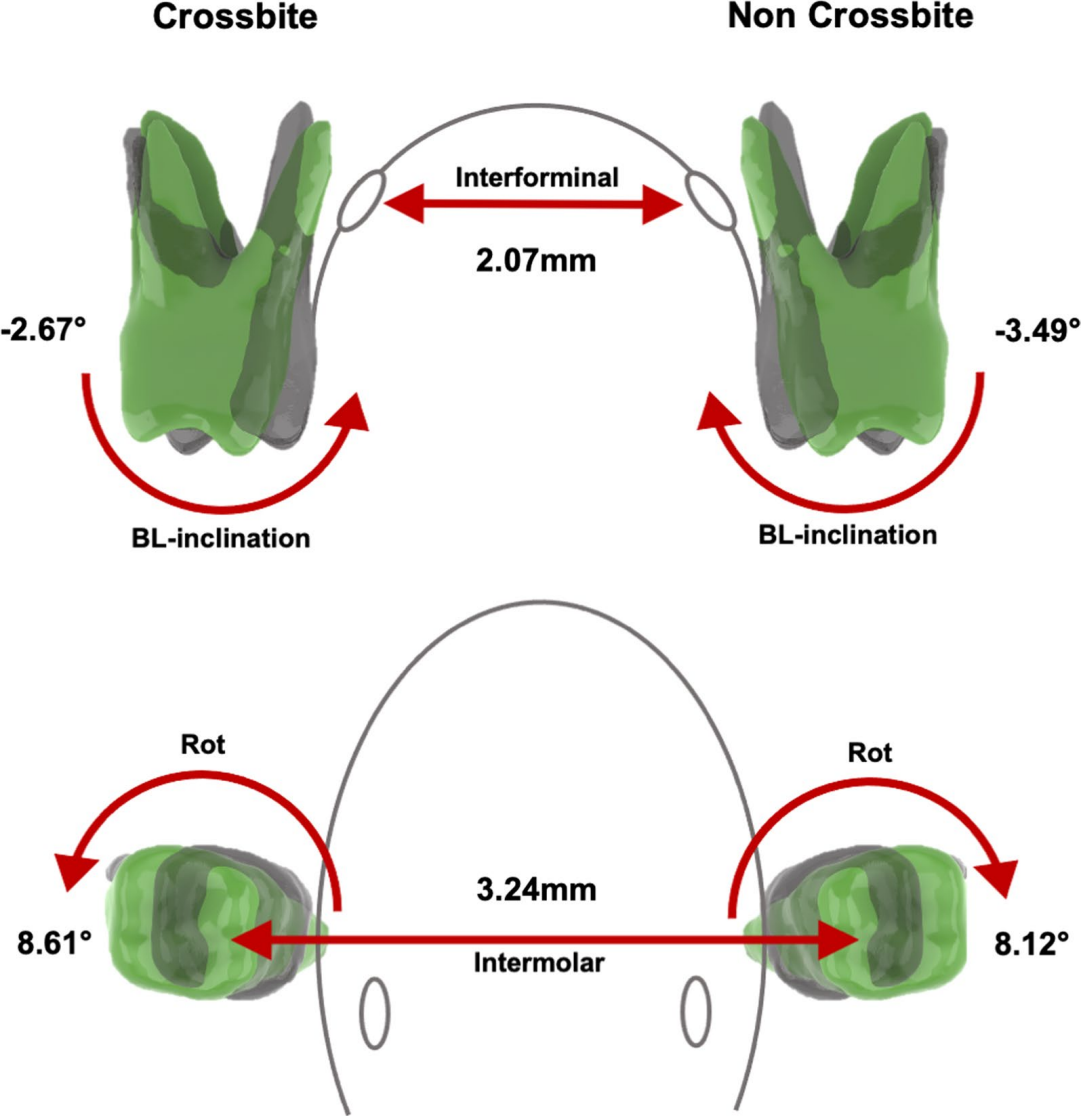
3D graphical representation of the statistically significant changes between T1 (green) and T2 (black) observed in the RME-E group

**Fig. 4:**
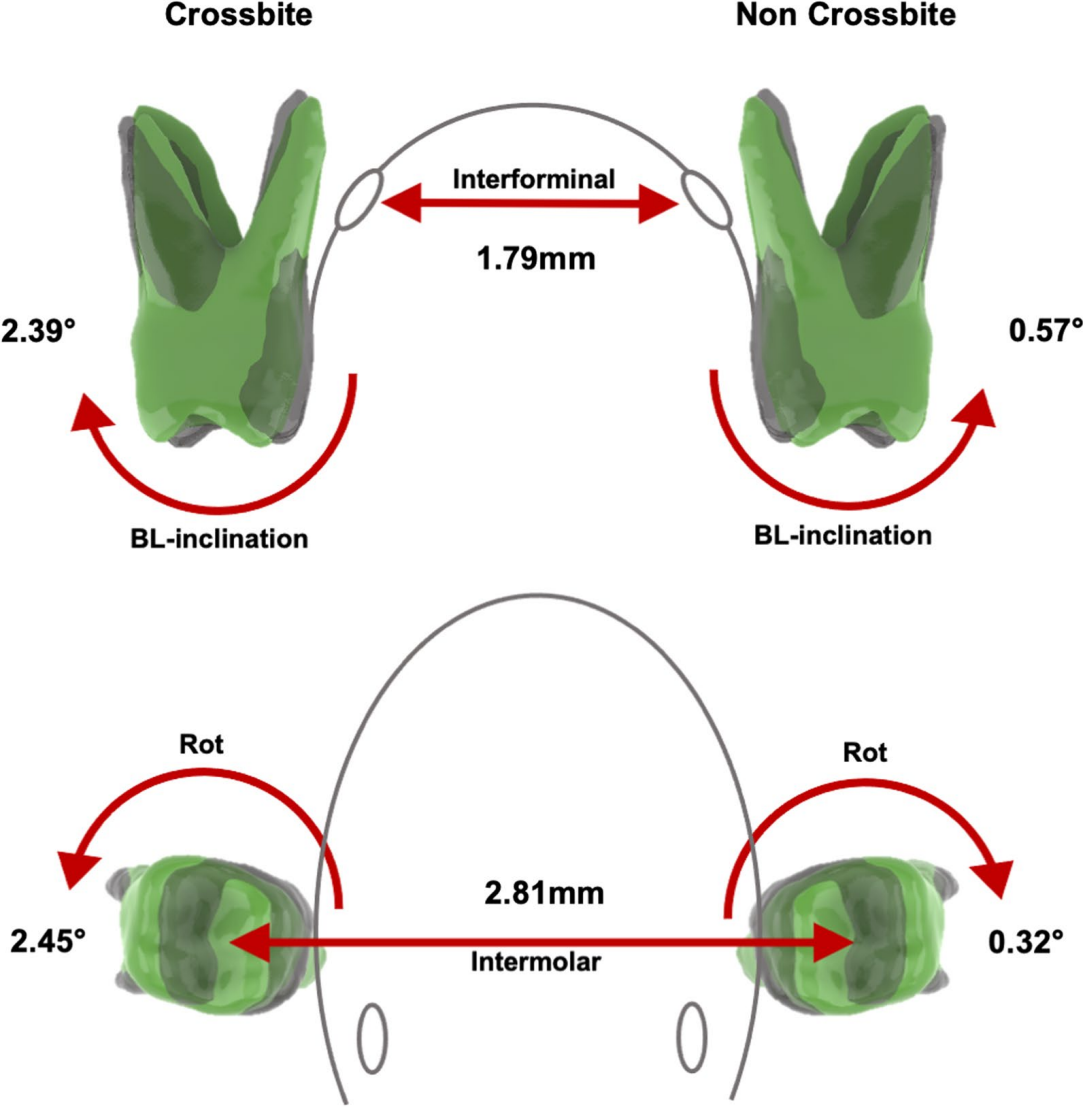
3D graphical representation of the statistically significant changes between T1 (green) and T2 (black) observed in the RME-6 group

Finally, Figs. 3 and 4 graphically summarized the most clinically and statistically significant changes in the RME-E and RME-6 groups, respectively.

## Discussion

Orthopedic forces exerted by the RME separate the maxillary basal bone, resulting in the opening of the midpalatal suture. The maxilla splits into right and left hemimaxilla by a lateral rotation which could influence increased values of buccal tipping and tooth rotation when the craniofacial complex is used as a reference. Therefore, the aim of the present prospective study was to compare the positional changes of the maxillary first molars after RME using deciduous or permanent teeth as anchorage with an intermaxillary reference system in three dimensions to reduce the bias of the measurements due to the three-dimensional movements of the maxillary bodies during treatment.

Both the RME-E and RME-6 groups reported a mean skeletal expansion of 2.07 mm and 1.79 mm, respectively; the corresponding values for the intermolar increase were 3.24 mm and 2.81 mm, with no significant differences between the two treatment modalities, indicating the efficacy of maxillary expansion therapy; in fact, the calculated ratio between skeletal and dental expansion (Interforaminal/Intermolar*100) are 63.9% and 63.7% for RME-E and RME-6 groups, respectively.

A previous study reported the biomechanics of bone distraction produced at cranial sutures by RME [19]. The expansion pattern of the maxilla can be divided into the rotation of the naso-maxillary complex and the buccolingual inclination of the teeth. When the maxillary bone is successfully transected by RME, the naso-maxillary complex splits transversely in a pyramidal-like configuration in coronal sections, with the center of rotation is in the fronto-nasal suture; that is inevitably accompanied by a change in the alveolar bone inclination as part of the rotational arm. Therefore, the greatest widening is observed in the dento-alveolar structures, with the expansion effect gradually decreasing in the upper structures. Moreover, full skeletal expansion is not achieved due to the resistance of the adjacent hard and soft structures surrounding the maxillary bones. The same pyramidal configuration with a fulcrum point into the pterygoid processes of the sphenoid bone minimizes the ability of the palatal bones to separate in the midsagittal plane and results in a greater opening of the anterior than posterior portion of the midpalatal suture in a ratio of approximately 3:2 [20]. Thus, the presence of an upper and posterior center of rotation could indirectly affect the measurement of buccolingual molar inclination or rotation, respectively. The authors call this “double drift effect”: teeth move into the maxilla while the maxilla moves relative to the cranial base. For this reason, bilateral measurements, i.e., measurements of the entire upper jaw, may be distorted. Thank you to 3D technology, the hemimaxillary plane has been mathematically constructed and provides a spatial reference that should not be influenced by previous effects, resulting in more reliable measurements.

The proper time for rapid maxillary expansion cis the early mixed dentition, after the first permanent molars have fully erupted. RME has been widely investigated, and many systematic reviews have been carried out on the effects of RME on dental and skeletal structures analyzed by 2D methods [1, 21–23]; it is known that treatment performed in the first transition period has been identified as a favorable condition for stability and has minimal adverse effects on the permanent dentition [11]. The literature reports that the mean expansion in the posterior midpalatal suture ranges from 0.84 to 2.88 mm, which corresponds to 12–52.5% of the screw expansion [24]. The rest of the expansion is caused by buccal bending of the alveolar structures and buccal tipping of the teeth, which occur under heavy expansion forces, also depending on the anchorage unit and the maturation stage of the midpalatal suture [25]. In addition, a percentage of relapse should always be expected with both anchorage solutions depending on the follow-up period and the device design. It is more important to understand the possibility of bias introduced by the orthopedic effect on the dental measurements.

In the present study no significance was found in the mesiodistal angulation of the first molars in both the RME-E and RME-6 groups, in contrast to the buccolingual inclination. The buccal inclination has been frequently investigated because of the periodontal side effects it may cause [26]. It has been suggested that prior to treatment, teeth on the side of crossbite usually have less buccal inclination, while teeth on non-crossbite side have greater buccal inclination due to of a physiological dental compensation that occurs spontaneously to achieve tooth occlusion. It has been reported that upper first molars, as anchorage teeth, may have a mean buccal inclination of 5° during expansion, which partially recedes during the retention phase [27]. In the present study, RME-E showed a decrease in buccolingual inclination in both crossbite and non-crossbite side compared to RME-6, which showed a slight increase in this variable, and these results were significant between groups. The buccal inclination of the maxillary first molars occurred in the RME-6 group, although the jackscrew was used very close to the palatal vault to apply the force closer to the center of resistance of the anchor teeth, thus preventing their buccal inclination. The increase in buccal inclination of maxillary permanent molars in the RME-6 group has already been reported by several authors [28] and is usually associated with the outward tilting of the alveolar process, which leads to bending of the alveolar bone, contributing to an increase in the buccal inclination measurement of the first molars [29]. On the contrary, when RME is performed with primary molars as anchorage, the permanent first molars could decrease buccal inclination, thus counteracting bone bending, since the occlusal contacts present during RME overcorrection (the palatal cusp of the maxillary permanent first molars occludes on the inner slope of the buccal cusp of the mandibular permanent first molars) could contribute to an occlusal adaptation that significantly decreases their buccal inclination, as shown by previous studies and consistent with the present results [17]. This occlusal adjustment can only occur if the maxillary first molars are not fixed in the appliance with bands or other auxiliaries [18]. Another important consideration relates to overcorrection expansion. Indeed, overcorrection contacts are usually highlighted by the buccal inclination of the maxillary first molars, which forces the clinician to interrupt screw activation to avoid a scissor bite, even though the extent of clinically required maxillary expansion may not be sufficient in terms of transversal deficits or crowding. Based on the present results, a lingual tilting of the maxillary first molars could occur when RME is performed on primary teeth, to counteract occlusal contacts leading to a scissor bite and then shift the overcorrection contacts to higher screw activation rates, when clinically required.

Another finding of the present study was that the upper first molars of RME-E exhibited significant distal rotation compared with RME-6. As mentioned earlier, the effects of RME include the triangular opening of the midpalatal suture, which could lead to distorotation of the posterior teeth. RME on permanent molars does not allow the anchorage teeth to adapt to the best occlusal situation because they are constricted with bands and the movement is limited to the deformation of the expander. RME on deciduous molars could lead to accommodation between upper and lower molars with distal rotation, resulting in an improvement of the occlusion in the class II and a less invasive second phase of treatment, as previously suggested [11, 30].

Lower arch changes were not evaluated in the present study and it might be considered as a limitation. Previous investigations suggested that the lower first permanent molars might play a role and undergo changes in inclination and rotation after changes to the upper first molars, leading to new a occlusal balance [18].

The use of CBCT in orthodontics allowed a changes in the perception of 3D craniofacial structures and a more accurate localization of these structures. Nevertheless, any reference system and landmark identification can be biased when there are a large number of variables and information. The main effort was to reduce the biases related to the minimal changes in the reference structures, which, according to previous studies, should not change significantly in the observed time intervals [31]. The dental pulp horns and root apexes could be carefully localized. The pulp chambers do not undergo significant changes, e.g., atresia and calcific metamorphosis, during the application of orthopedic force, although they serve as anchor teeth, as reported [32]. Also, the magnitude of RME forces on immature roots appears to result in short-term interruption of root development without apical resorption of the mature tooth apex [33]. In addition, measurements were made in two halves of the maxilla to avoid bias in terms of overall diameter, suggesting that the crossbite and noncrossbite sides might undergo different changes. This could be considered one of the most important innovations of the present study.

### Limitations

The main limitation of the present study is the small sample size and the impossibility to subdivide it further than the presence or absence of crossbite. Also for this reason, it is suggested, when clinically feasible, to use split-mount studies to evaluate changes independently from the different growth patterns among various groups.

## Conclusions

Based on the findings that we have reported, the following conclusions can be drawn:

1. Regardless of the anchorage teeth, RME is effective in the orthopedic treatment of transverse maxillary deficiency, although dental effects are observed to varying degrees;
2. In RME with anchorage to maxillary permanent first molars, no significant movement of this teeth was observed, except for buccal inclination on the crossbite side, which, however, is of little clinical significance;
3. RME with anchorage to deciduous molars spontaneously resulted in significant lingual inclination and distal rotation of maxillary first molars when not forced into the appliance and free to adapt to the occlusal contacts.

## Data Availability

The datasets generated and/or analyzed during the current study are not publicly available due privacy limitations but are available from the corresponding author on reasonable request.

## Abbreviations

RME: Rapid maxillary expansion
CBCT: Cone beam computed tomography
